# Flexible 5-in-1 Microsensor Embedded in the Proton Battery for Real-Time Microscopic Diagnosis

**DOI:** 10.3390/membranes11040276

**Published:** 2021-04-08

**Authors:** Chi-Yuan Lee, Chia-Hung Chen, John-Shong Cheong, Yun-Hsiu Chien, Yi-Chuan Lin

**Affiliations:** 1 Department of Mechanical Engineering, Yuan Ze Fuel Cell Center, Yuan Ze University, Taoyuan 32003, Taiwan; johnshong1018@gmail.com (J.-S.C.); s1040923@g.yzu.edu.tw (Y.-H.C.); chuan881018@gmail.com (Y.-C.L.); 2 HOMYTECH Global Co., Ltd., Taoyuan 33464, Taiwan; chenjahon@gmail.com

**Keywords:** proton battery, flexible 5-in-1 microsensor, real-time microscopic diagnosis

## Abstract

The proton battery possesses water electrolysis, proton storage and discharging functions simultaneously, and it can be manufactured without expensive metals. Use the principle of proton exchange membrane water electrolysis for charging, store it in the activated carbon on the hydrogen side and use the principle of proton exchange membrane fuel cell for discharge when needed. According to the latest literature, it is difficult to obtain the exact important physical parameters inside the proton battery (e.g., voltage, current, temperature, humidity and flow), and the important physical parameters are correlated with each other, which have critical influence on the performance, lifetime and health status of the proton battery. At present, the condition of the proton battery is judged indirectly only by external measurement, the actual situation inside the proton battery cannot be obtained accurately and instantly. Therefore, this study uses micro-electro-mechanical systems (MEMS) technology to develop a flexible 5-in-1 microsensor, which is embedded in the proton battery to obtain five important physical parameters instantly, so that the condition inside the proton battery can be mastered more precisely, so as to prolong the battery life and enhance the proton battery performance.

## 1. Introduction

The proton battery is a very novel research area, first published by Heidari et al. [1] from the Royal Melbourne Institute of Technology (RMIT), Australia, March 2018. It is rechargeable, and it can electrolyze water, the electrolyzed protons can be stored in activated carbon, and the protons can be released and combined with oxygen to generate electricity. However, the current overall output performance is obtained by externally measuring the proton battery, so the accurate information inside the proton battery cannot be known instantly. The proton battery is built on the proton exchange membrane fuel cell (PEMFC), the major difference between them is that the hydrogen of the proton battery is stored in the battery in the state of ions, not outside the battery. This innovative breakthrough can reduce the battery cost and the space occupied by the hydrogen tank greatly, and the application area is enlarged. 

At present, the fuel cell technology has been used in different areas extensively, but there are still many problems to be solved, such as the fuel cell volume, weight and hydrogen storage problems. In comparison to PEMFC, the proton battery is rechargeable and free of hydrogen; the hydrogen ions will be released from the battery. In the charging process, the proton battery can decompose water with the assistance of power supply to generate hydrogen ions, which are stored in the porous carbon-based material as a hydrogen storage electrode. Folonari and Condon et al. [2,3] thought that the new kind of fuel cell composed of hydride electrode and solid electrolyte had higher energy density and a more compact simple structure, and, if it maintained the advantages of a traditional fuel cell, this structure could enhance the reliability and performance of electric vehicles significantly. At present, the mainstream fuel cells mostly store the hydrogen in the hydrogen storage tank and the hydrogen storage tank is installed on the carrier (e.g., automobile and generator). Most hydrogen storage tanks use metal hydride technology to store hydrogen; the idea was first proposed by Andrews et al. In the charging mode of the unitized regenerative fuel cell using the metal hydride-nitride composite electrode, the hydrogen storage in the electrode is 0.6 wt% and the emission of hydrogen is detectable, but very low, only about 0.01 wt%, inducing a safety risk [4]. Jurewicz et al. [5,6,7] first pyrolyzed the viscose fabric of the liquid KOH electrolyte (concentration 6M) to prepare an activated carbon electrode for electrochemical hydrogen storage, which implemented 1.5 wt% reversible hydrogen storage, and studied the influence of the acidity and alkalinity of the electrolyte on hydrogen storage capacity. Babel et al. [8] fabricated a highly porous carbon; the 1.89 wt% high hydrogen storage capacity proved again that ultramicropore and micropore volume and relatively smaller mesopore volume enhanced the hydrogen storage capacity. Bosch et al. [9] enlarged the surface area and improved the material. As the hydrogen storage characteristic of carbon nano-structure was understood, the feasibility of the rechargeable the proton battery with high energy density and high hydrogen storage capacity could be enhanced. The proton battery is a very novel research area, there are many immature prototypes [10,11,12] worth further research. 

Zhang et al. [13] found that when the immersion ratio was 4.5:1 (KOH/char), the activation temperature of activated carbon was 800 °C, the activation time was 1.5 h and the maximum surface area ratio was 2388 m^2^g^−1^. Li et al. [14] used six kinds of sulfur-activated carbon with different specific surface areas to study the influence of electrochemical properties and specific surface area on electrochemical properties. When the current density in 6M KOH was 0.05 Ag^−1^, the capacitance value was 395 Fg^−1^. 

Laribi et al. [15] found that when the working temperature of the fuel cell rose from 60 to 140 °C, the resistance of the thin film rose with time, and the water content in the thin film decreased, so the ionic conductivity decreased. 

Yang et al. [16] used platinum (Pt) as a thermistor, which was deposited on the film surface; the surface temperature was calculated according to the resistance temperature correction data. 

Ko et al. [17] studied the influence of working pressure on the fuel cell performance. The findings showed that when the relative humidity was low, the influence of water delivery was more important than reaction kinetics. The water flow could be controlled by adjusting the working pressure to obtain appropriate water distribution and optimum performance. 

Sun et al. [18] used micromachining technology to manufacture a flow sensor and studied the influence of different geometric parameters on temperature difference. 

Aslam et al. [19] probed into the effect of different flow rates on the cell. The performance of the fuel cell declined as the flow increased. In addition, the speed of removing water from the cathode increased with air flow rate, so that the moisture for humidifying the membrane electrode assembly decreased. 

**Figure 1:**
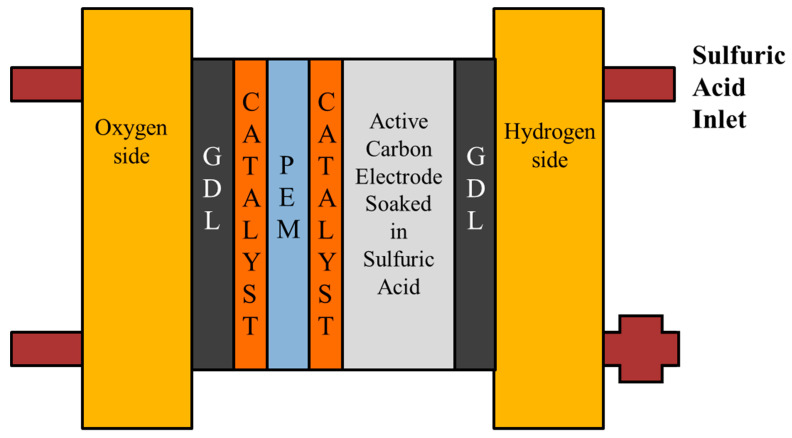
Schematic diagram of the proton battery.

This study uses MEMS technology to develop an internal diagnostic tool for the proton battery, which is combined with a flexible 5-in-1 microsensor and embedded in the proton battery for measuring the five important physical parameters simultaneously, so that the actual situation inside the proton battery can be mastered more accurately, so as to enhance the proton battery performance. Its internal structure is shown in Figure 1. The principle of use of the proton battery in the oxygen side provides water for water electrolysis, and the electrolyzed hydrogen protons move through the proton exchange membrane to the hydrogen side and are stored in activated carbon. When discharging, the oxygen side provides oxygen and the hydrogen side releases hydrogen protons for fuel cell reaction. In this study, the flexible 5-in-1 microsensor is moved into the hydrogen side upstream, the oxygen side upstream and the oxygen side downstream of the proton battery, and the internal voltage, current, temperature, flow and battery are measured when the proton battery is charged. When discharging, the humidity of the gas inside.

## 2. Flexible 5-in-1 Micro-Sensor Production Process

**Figure 2:**
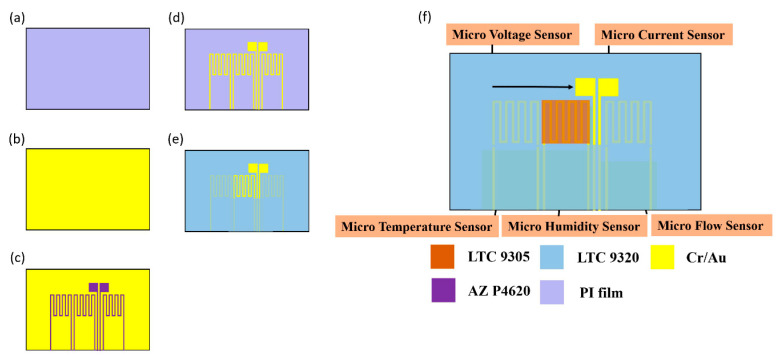
Schematic diagram of process of the flexible 5-in-1 microsensor.

This study used MEMS technology to integrate five sensing structures, including temperature, voltage, current, flow and humidity. The process of the flexible 5-in-1 microsensor is shown in Figure 2.

**Figure 3:**
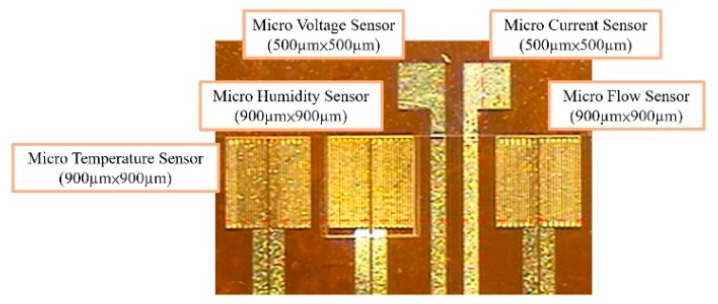
Optical micrograph of the flexible 5-in-1 microsensor.

(a) First, the PI (polyimide) film is cleaned with acetone and methanol organic solutions respectively, the residual methanol is removed by DI (deionized) water, and the surface dust, residual oil and fat are removed, so as to enhance the adhesion of the thin film metal.
(b) The Cr and Au are evaporated by E-beam evaporator as the adhesion layer and sensing electrode layer.
(c) The patterns of micro temperature, voltage, current, flow and humidity sensors are defined by photolithography.
(d) The Au etching solution and Cr etching solution are used for wet etching.
(e) LTC 9320 is coated as a protection layer, and the sensing areas and pins of micro voltage, current and humidity sensors are defined by the photolithography process, which are exposed and not covered with a protection layer.
(f) LTC 9305 is coated as the humidity sensitive thin film of the micro humidity sensor and the process of the flexible 5-in-1 microsensor is completed. Figure 3 shows the optical micrograph of the flexible 5-in-1 microsensor.

## 3. Correction of the Flexible 5-in-1 Microsensor

The micro voltage and current sensors directly export the voltage and current from the proton battery for detection, thus additional correction is unnecessary. The correction of micro temperature, humidity and flow sensors is described below.

### 3.1. Temperature Correction of Flexible 5-in-1 Microsensor

**Figure 4:**
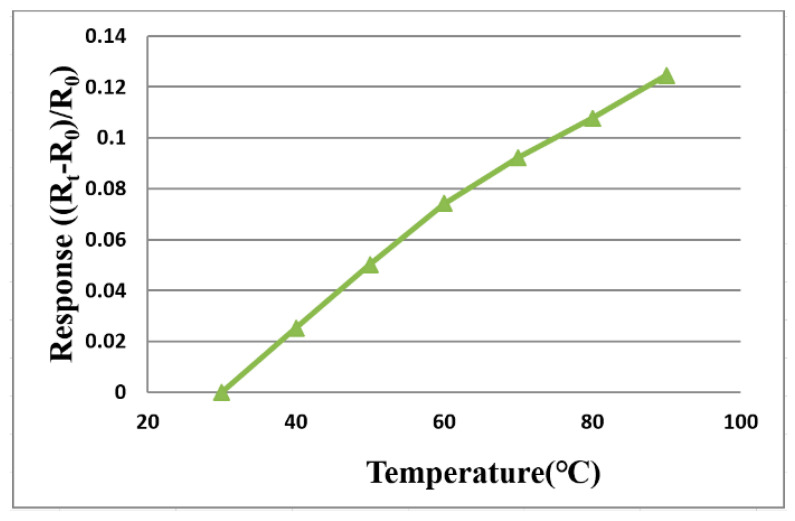
Correction curve of the micro temperature sensor.

The flexible 5-in-1 microsensor and the thermometer of the BM-525 BRYMEN digital multimeter are put in the DENG YNG DS45 Drying Oven, the reference is set up and the temperature is stabilized, then the resistance value of the micro temperature sensor is extracted. In the working temperature range, the resistance value is extracted at intervals of 10 °C, the micro temperature sensor is corrected three times, the average value is taken, and the measured correction curve approximates linear variation, as shown in Figure 4. 

### 3.2. Humidity Correction of the Flexible 5-in-1 Microsensor

**Figure 5:**
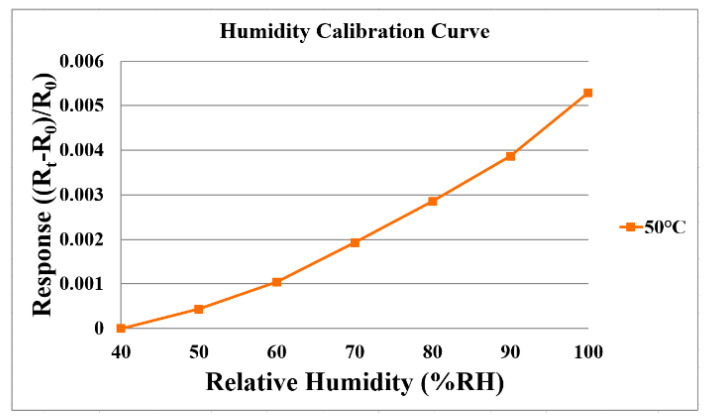
Correction curve of micro humidity sensor.

For humidity correction, the constant temperature and humidity testing machine is used as environmental criteria, from relative humidity 40% to 100%, and the temperature is fixed in the operating state. Each time when the relative humidity recording point is increased, the heater on the micro humidity sensor is heated to evaporate the residual moisture at the previous recording point. Each time after 120 min of stabilization, the NI PXI 2575 data acquisition unit is used to extract the resistance value of the micro humidity sensor instantly, so as to obtain the correction curve. Figure 5 shows the average correction curve of the micro humidity sensor; the higher the temperature is, the larger the humidity variation is.

### 3.3. Flow Correction of the Flexible 5-in-1 Microsensor

When charging, it is necessary to provide water on the oxygen side to produce water electrolysis. The hydrogen side produces hydrogen protons and hydrogen. When discharging, oxygen is provided. Therefore, the water flow and oxygen flow must be corrected.

**Figure 6:**
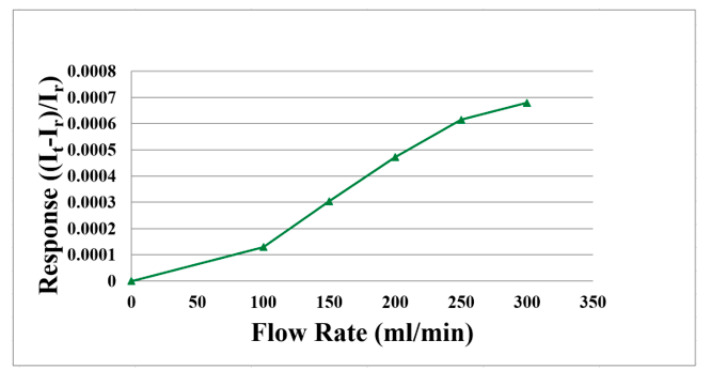
Average correction curve of the micro flow sensor (H_2_).

**Figure 7:**
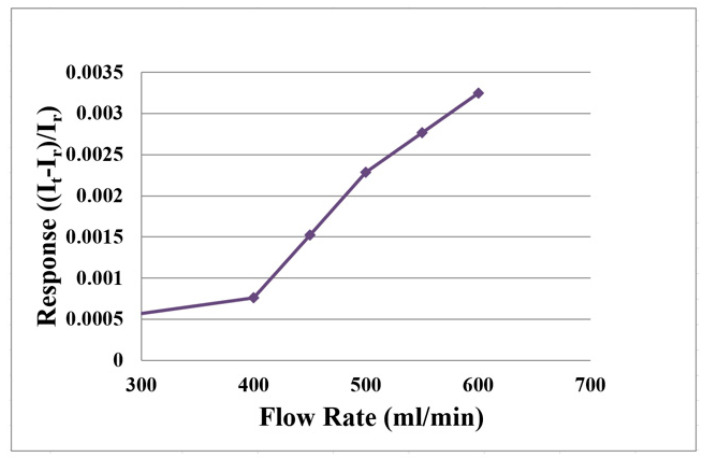
Average correction curve of the micro flow sensor (O_2_).

The flexible 5-in-1 microsensor is embedded in the proton battery and the battery testing machine supplies gases (H_2_ and O_2_) as the source of flow. The power supply is connected to the signal pin of micro flow sensor and the anode is connected to BM-525 BRYMEN digital multimeter in series to measure the current variation. The power supply supplies constant voltage to the micro flow sensor to generate a stable heat. First, the reference current value at 0 mL/min is measured; the hydrogen flow correcting range is 100 to 300 mL/min, it is measured at intervals of 50 mL/min. The oxygen flow correcting range is 400 to 600 mL/min, it is measured at intervals of 50 mL/min. The micro flow sensor is corrected and the average value is taken; the measured average correction curve is shown in Figure 6 and Figure 7. 

**Figure 8:**
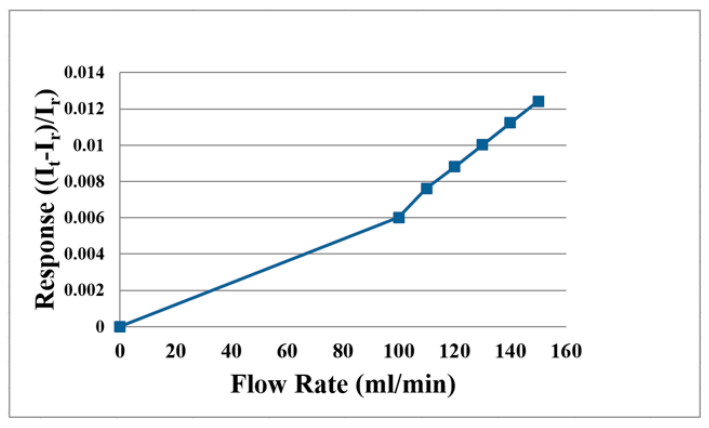
Average correction curve of the micro flow sensor (H_2_O).

For liquid (H_2_O) flow correction, the LEADFLUID BT100S-1 speed adjusting peristaltic pump provides steady flow. The flow correction range is 100 to 150 mL/min, measured at intervals of 10 mL/min; the average correction curve of the micro flow sensor is shown in Figure 8. 

### 3.4. Thermal Shock Test for the Flexible 5-in-1 Microsensor

There is an electrochemical reaction inside the proton battery during charging or discharging, this variation generates heat energy and temperature. Therefore, the thermal shock test is performed for the flexible 5-in-1 microsensor with relatively strict internal ambient temperature, so as to test whether the flexible 5-in-1 microsensor can maintain its performance as the temperature changes rapidly. 

The corrected flexible 5-in-1 microsensor is put in the thermal shock machine and the preset temperature is changed quickly to simulate the harsh environment inside the proton battery. After repeated thermal shock tests, the flexible 5-in-1 microsensor is corrected again, and the correction data before and after thermal shock are compared to check whether the linear curves are coincident or not. 

**Figure 9:**
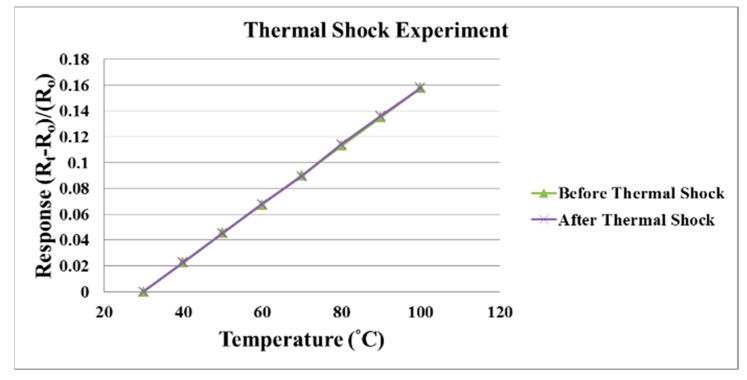
Correction curves before and after the thermal shock test for the flexible 5-in-1 microsensor.

The thermal shock test parameters are shown in Table 1. The thermal shock experiment is performed in the temperature range of 30 to 100 °C and the heating rate is 35 °C/min. When the temperature is reduced from 100 to 30 °C, the declining temperature rate is −35 °C/min. This process is repeated 40 times. There is no exception found in the flexible 5-in-1 microsensor correction curves before and after the thermal shock test, proving the reliability of the flexible 5-in-1 microsensor, as shown in Figure 9.

## 4. Internal Real-Time Microscopic Diagnosis of the Proton Battery

**Figure 10:**
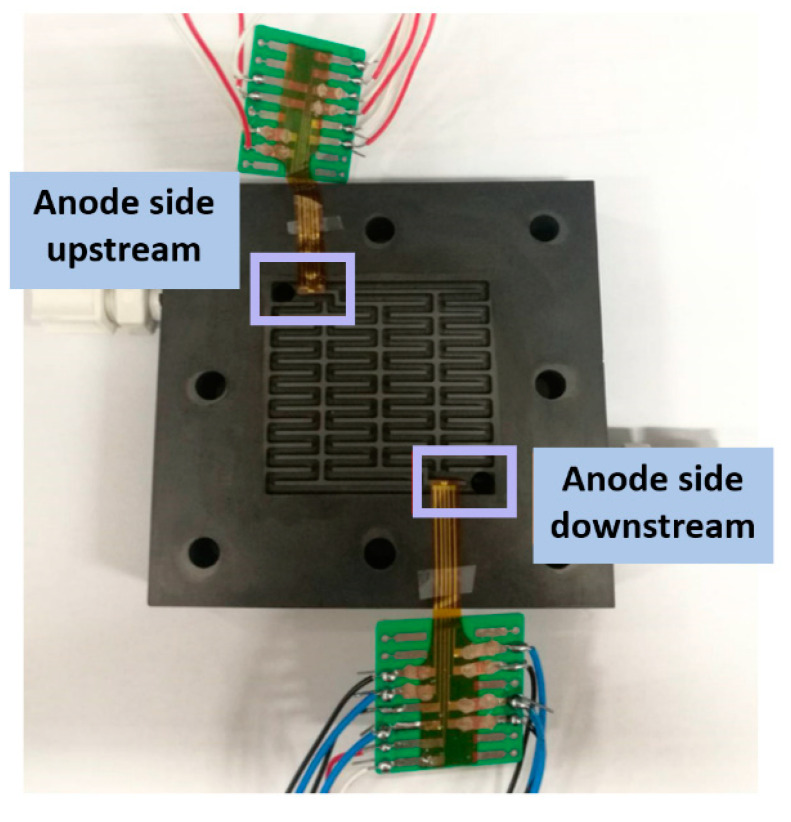
The flexible 5-in-1 microsensor embedded in the anode position.

**Figure 11:**
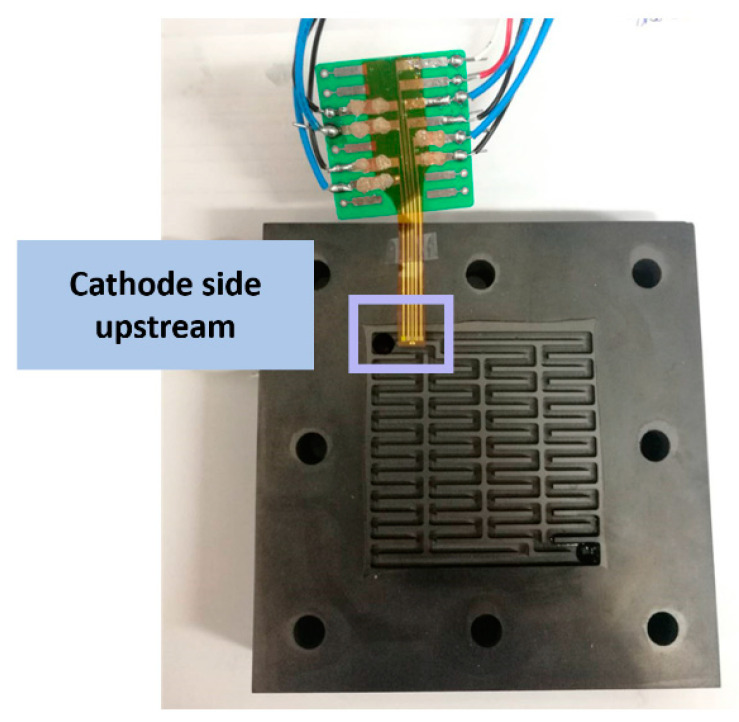
The flexible 5-in-1 microsensor embedded in the cathode position.

The proton battery technology is built on the fuel cell, so their materials are about the same. The difference is the intermediate carbon layer, whereby the BET (Brunauer–Emmett–Teller) area size can influence the hydrogen storage capacity of the proton battery. The activated carbons from two companies are studied and it is found that simply cleaning the activated carbon cannot improve the BET area. However, when different concentration ratios of KOH are heated to 800 °C and activated, the BET area can be enlarged a lot. The findings show that the activated carbons from different companies have different KOH concentration ratios for generating maximum BET area, and the BET area decreases when the concentration ratio producing the maximum value is exceeded. Finally, the KOH concentration ratio of 3:1 of Company A is used for the subsequent experiment. Figure 10 and Figure 11 show the flexible 5-in-1 microsensor embedded in the anode and cathode positions of the proton battery, respectively. 

### 4.1. Voltage Distribution Inside the Proton Battery 

**Figure 12:**
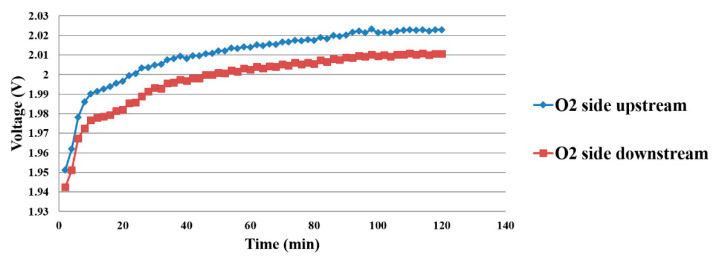
Current data of power supply.

As the voltage measurement must be connected to the hydrogen side, the micro voltage sensor on the hydrogen side is connected to the micro voltage sensor on the oxygen side. When the proton battery is being charged, the upstream and downstream microsensor voltage variations on the oxygen side are shown in Figure 12.

### 4.2. Current Density Distribution Inside the Proton Battery

**Figure 13:**
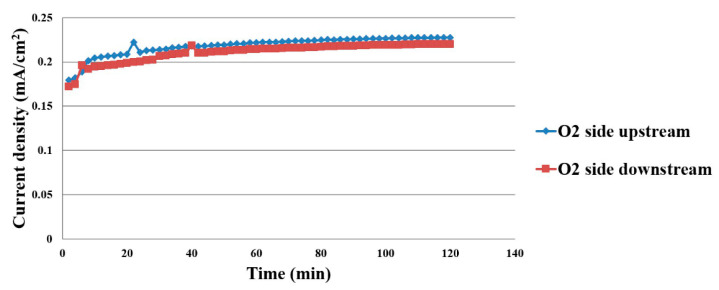
Current density distribution inside the proton battery.

The data measured by micro current sensor have the same uptrend as the micro voltage sensor does, thus it can be deduced that the proton battery is in a stable electrochemical reaction state, as shown in Figure 13. 

### 4.3. Temperature Distribution Inside the Proton Battery

**Figure 14:**
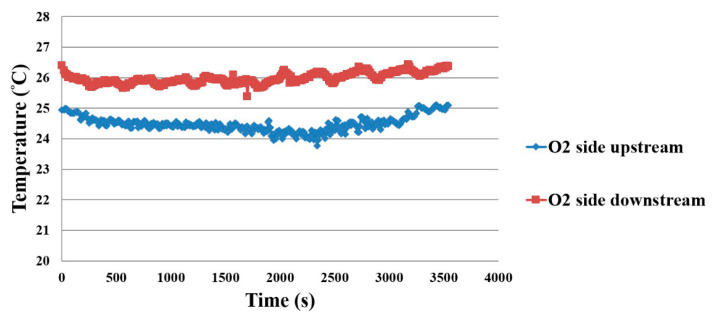
Oxygen side temperature distribution inside the proton battery.

**Figure 15:**
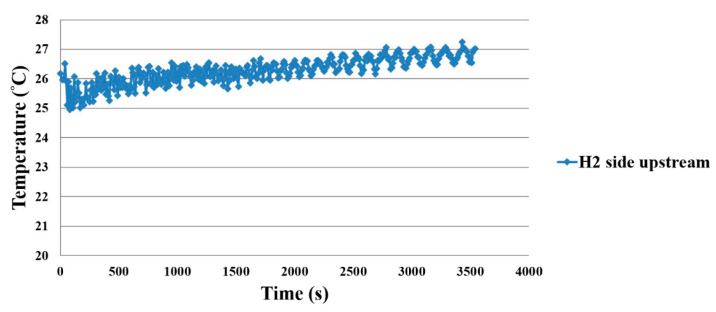
Hydrogen side temperature distribution inside the proton battery.

When the working temperature is 25 °C, the microsensor is placed in different positions on the oxygen side and hydrogen side, so as to observe the temperature distribution of the proton battery, as shown in Figure 14 and Figure 15. The water inlet is in the upstream position, so the upstream temperature is lower, and the downstream temperature is higher. 

### 4.4. Flow Distribution Inside the Proton Battery

**Figure 16:**
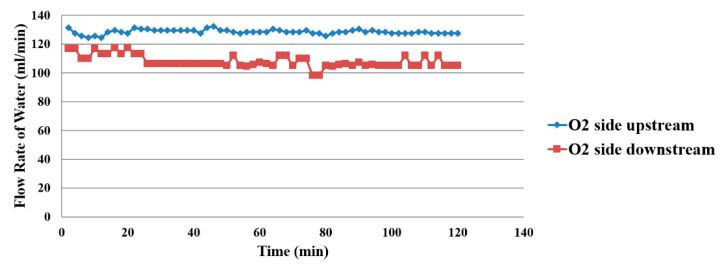
Oxygen side flow distribution inside the proton battery.

**Figure 17:**
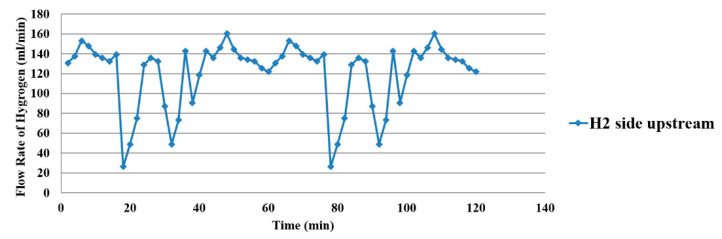
Hydrogen side flow distribution inside the proton battery.

The water flow distribution on the oxygen side is shown in Figure 16. The upstream oxygen side has a higher flow than downstream, as the water electrolysis and gas diffusion layer inside the proton battery obstruct the flow of water. Only the hydrogen flow was measured on the hydrogen side, as shown in Figure 17. 

### 4.5. Humidity Distribution Inside the Proton Battery

**Figure 18:**
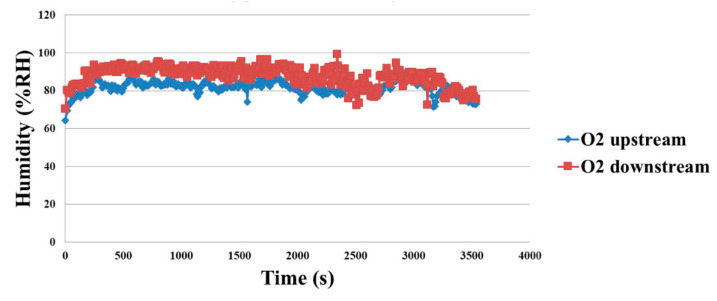
Oxygen side humidity distribution during discharging.

As shown in Figure 18, the oxygen side increases from lower humidity, and the downstream humidity is higher, this is related to the combination of hydrogen and oxygen. As the electricity generation decreases, the reaction is slighter, and the upstream and downstream humidity overlap each other in the late stage. 

## 5. Conclusions

The flexible 5-in-1 microsensor is embedded in the proton battery for internal real-time monitoring, whereby five physical quantities can be measured at the same time. This flexible 5-in-1 microsensor has five sensing functions (voltage, current, temperature, flow and humidity), characterized by resistance to electrochemical environment, small thickness, small area, high sensitivity, real-time measurement and arbitrary placement in the proton battery. Furthermore, it is flexible and unlikely to be damaged. It has greater development potential in the future commercialization of the proton battery. The measurement of the microsensor in the proton battery is in line with expectations. It can detect local physical quantities and allows the use of these data to adjust the membrane to extend the life and performance of the membrane.

## Figures and Tables

**Table 1:** Thermal shock test parameters.

Schedule	Process	Set Time
Preparation	The temperature of the thermal shock machine is preheated to 30 °C	-
Step 1	30 °C to 100 °C	2 min
Step 2	Constant temperature time	13 min
Step 3	30 °C	2 min
Step 4	Constant temperature time	13 min
Done	30 °C ~ Room temperature	-
Cycle: 40		1 Cycle = 30 min
